# Girls’ hidden penalty: analysis of gender inequality in child mortality with data from 195 countries

**DOI:** 10.1136/bmjgh-2018-001028

**Published:** 2018-10-30

**Authors:** Neelam Iqbal, Anna Gkiouleka, Adrienne Milner, Doreen Montag, Valentina Gallo

**Affiliations:** 1 BSc in Global Health, Centre for Primary Care and Public Health, Queen Mary, University of London, London, UK; 2 Department of Sociology, University of York, York, UK; 3 Centre for Primary Care and Public Health, Queen Mary, University of London, London, UK; 4 Epidemiology and Medical Statistics, London School of Hygiene and Tropical Medicine, London, UK; 5 School of Public Health, Imperial College London, London, UK

**Keywords:** gender inequality, gender inequality index (GII), child mortality, child mortality sex ratio, ecological analysis

## Abstract

**Introduction:**

Gender inequality has been associated with child mortality; however, sex-specific mortalities have yet to be explored. The aim of this study is to assess the associations between gender inequality and the child mortality sex ratio at country level, worldwide and to infer on possible mechanisms.

**Methods:**

Data on sex-specific under-five mortality rates (U5MR) and the corresponding sex ratio (U5MSR) for the year 2015, by country, were retrieved from the Unicef database. Excess under-five female mortality was derived from previous published work. Gender inequality was measured using the Gender Inequality Index (GII). Additional biological and social variables have been included to explore potential mechanistic pathways.

**Results:**

A total of 195 countries were included in the analysis. In adjusted models, GII was significantly negatively associated with the U5MSR (β=−0.29 (95% CI −0.42 to –0.16), p<0.001) and borderline significantly positively associated with excess under-five female mortality (β = 3.25 (95% CI −0.28 to 6.67, p=0.071). The association between GII and U5MSR was strong and statistically significant only in low-income and middle-income countries and in the Western Pacific area.

**Conclusion:**

The more gender unequal a society is, the more girls are penalised in terms of their survival chances, in particular in low-income and middle-income countries. In order to decrease child mortality and excess girl mortality, global policy should focus on reducing gender inequality surrounding measures of reproductive health, women’s political empowerment, educational attainment and participation in the workforce.

**Key questions**

**What is already known?**

- Gender inequality is associated with increased child mortality rates at country level, worldwide, potentially through mechanisms relating to child’s capability of survival or maternal factors impacting child’s survival.
- However, no study has investigated if gender inequality is also associated with a sex differential in child mortality.

**What are the new findings?**

- Worldwide, gender inequality not only is associated with increased under-five mortality rates, but girls seem to disproportionally suffer from this, particularly in lower-income and upper-middle-income countries.
- Only biological factors reflecting the living conditions of children under 5 years are independently associated with under-five mortality sex ratio where other social or maternal conditions do not predict the under-five mortality sex ratio in multivariable models.

**What do the new findings imply?**

- In order to decrease child mortality rates, global policy should integrate a gendered perspective that addresses gender inequality not only in the field of reproductive health but also in terms of distribution of social determinants of health like women’s political empowerment, educational attainment and participation in the workforce.

## Introduction

Child and infant mortality varies across regions of the world: a total of 5.9 million children under the age of 5 years died in 2015.^1^ More than half of these early deaths are due to preventable diseases or treatable conditions, and thus, they are the result of socioeconomic marginalisation and of relative barriers in access to simple, affordable healthcare.^1^ It is worth noting that approximately 45% of these deaths are associated with malnutrition. Social determinants, as the combined structural forces such as gender, race, education and income, that impact on people’s lives play a critical role in child and infant mortality rates.^2 3^ Globally, children are at a greater risk of dying before age 5 if they are born in rural areas, within poor households and/or to a mother denied basic education.^4^

Mother’s education as a risk factor of child mortality mirrors the importance of gender as a social determinant of health.^5^ Gender impacts access to social power and distribution of resources, and it is intertwined with a number of cultural understandings relevant to men’s and women’s behaviour. Thus, gender is associated with a variety of both positive and negative health outcomes through a multitude of pathways. Those can be either direct, as in the example that women are more often victims of domestic violence,^6^ or indirect, affecting other social determinants of health like income.^7^

In this light, gender inequalities in health and mortality have been the subject of extensive research in the field of public and global health. Studies are broadly separated into two streams. The first stream seeks to explain these inequalities on the base of biological, psychological and social differences between men and women. The second one, developed mainly by feminist scholars, perceives gender health inequalities as one of the manifestations of overall gender inequality produced by dominant patriarchal and sexist ideologies. This renders girls’ and women’s lives less valued than boys’ and men’s^8 9^ and results in males benefiting over females across multiple domains since birth.^10^ Within this stream, the health and mortality impact of gender inequality and patriarchy has been repeatedly interrogated and it has been found to negatively affect health across the life course and mortality in both men and women^11^ although the latter are more often than not disproportionately affected.^5 6 10^ Situated in this tradition, the current study focuses on the relationship between gender inequality, as assessed by the Gender Inequality Index (GII), and mortality rates within a particularly and globally vulnerable population group, namely children under the age of five.

The GII measures gender inequalities in three aspects of human development: reproductive health, political empowerment and economic status. As such, the GII reflects the human development costs of gender inequality. A recent study investigated the association between the GII and low birth weight, child malnutrition and child mortality in 96 countries finding that 41% of the variance in child mortality is related to GII, when adjusting for Gross Domestic Product (GDP).^12^ This confirmed previous evidence of a positive correlation between GII and neonatal, infant and under-five mortality.^13^ However, sex-specific mortality rates have yet to be explored. This would allow an understanding of how gender inequality in different societal domains may perpetuate itself and whether and to what extent it is associated with subsequent gendered mortality inequalities, in terms of girls’ survival chances compared with their male counterparts.

The particular aims of this study are (1) to assess the associations between gender inequality and under-five mortality (for both boys and girls) and the under-five mortality sex ratio at country level, worldwide; (2) to infer on possible mechanisms underlying these associations (social and/or biological); and (3) to assess the interaction of these associations with categories of country income levels and regions of the world.

## Methods

### Data collection

In order to conduct an ecological analysis, all data at country level at specific time points, using publicly available data repositories, were collected.

Estimates of sex-specific under-five mortality rates (U5MR) and the sex ratio (U5MSR) for the year 2015, by country, were retrieved from the Unicef database.^14^ U5MR expresses the probability of dying before reaching 5 years of age, expressed per 1000 live births, and it was computed separately for males and females generating the male child mortality rate (MCMR) and female child mortality rate (FCMR). The M:F ratio of the U5MR indicated how many boys die for each girl within the same lifespan, generating the U5MSR: the higher the U5MSR, the higher the male mortality is compared with female, and vice versa. For each country, the excess female under-five mortality (per 1000 live births) in 2012 was also extracted from the work of Alkema and colleagues.^15^ This measured the difference between the expected female mortality rate in each country, which partially depends on total U5MR, and the observed rate for that country-year (where negative outcomes refer to lower-than-expected female mortality).^15^ Therefore, a negative excess female mortality refers to the lower-than-expected female mortality.

Gender inequality within each country was measured using the GII in 2015, available from the United Nation data repository.^16^ GII combines three aspects of human development: reproductive health (measured by maternal mortality ratio and adolescent birth rates), political empowerment (measured by proportion of parliamentary seats occupied by females and proportion of adult females with at least some secondary education) and economic status (measured by labour force participation rate of female and male population). The GII can vary between 0 and 1, with higher GII values showing greater gender inequality in a given country.

In order to explore to what extent potential associations could be explained by biological or socioeconomic factors, a number of additional variables have been considered. U5MR per 1000 live births due to acute respiratory infections and diarrhoea in 2015 were extracted from the Unicef data repository^17 18^ in order to explore potential biological mediating factors. Conversely, potential socioeconomic mediators were explored using the GDP per capita, and basic sanitation available in the country. GDP per capita in 2015 extracted from the World Bank data repository^19^ was defined as the GDP divided by the midyear population, and used to classify nations into low (below $1005), lower-middle ($1006–39 655), upper-middle ($3956-12 235) and high income ($12 236+) countries. The proportion of population using at least basic sanitation services (rural and urban combined) in 2015 was extracted by the WHO data repository.^20^ Finally, in order to explore to what extent potential association between gender inequality and child mortality is mediated by maternal access to perinatal care, information on the proportion of births attended by skilled personnel in years 1998–2016 was extracted by the WHO data repository.^21^

### Statistical analysis

The distribution of all variables was checked, and where not normal, the variables were log-transformed. The associations between GII and other potential mediating factors in relation to MCMR, FCMR, U5MSR and excess female mortality were assessed using linear regression models. Multivariable linear regression models were run to predict MCMR, FCMR, U5MSR and excess female mortality adding all potentially confounding variables into the model to explore the role of potential biological (U5MR due to acute respiratory infections and diarrhoea) or social mechanisms (GDP per capita, and basic sanitation) in the main associations.

In order to explore potential intersections with other social mechanisms, and the interplay between structural factors at country level, the interactions between GII and categories of GDP per capita groups and WHO regions (African, Americas, Southeast Asia, European, Eastern Mediterranean and Western Pacific Region) were tested, using the likelihood ratio test comparing two models with and without the interaction term. Where appropriate, associations were described by category.

## Results

**Figure 1:**
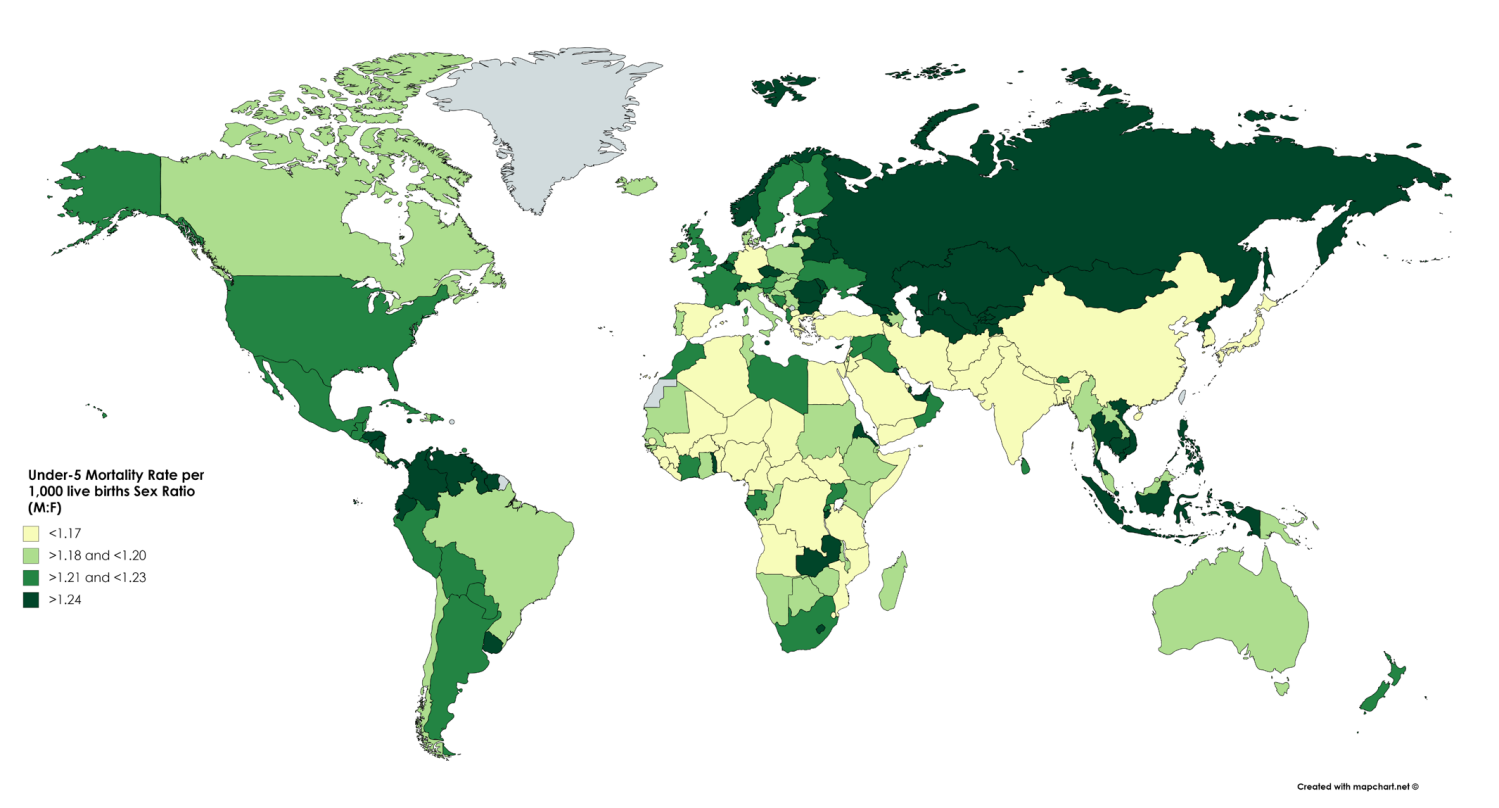
World map showing the under-five mortality rate per 1000 live births Sex ratio (U5MSR), adapted from Unicef.^14^ Categories calculated according to quartiles of distribution.

A total of 195 countries, worldwide, were included in the analysis. Higher male to female under-five mortality was observed in all countries, except for Tonga and India, where U5SR was 0.81 and 0.94, respectively. The U5MSR was the highest in Mongolia (1.48), Turkmenistan (1.44) and Viet Nam (1.40). Overall, the mean U5MSR was 1.20 (SD 0.07) and the median was 1.20 (IQR 1.17–1.23); U5MSR was higher in upper-middle and high-income countries, but a clear pattern among countries worldwide was not immediately evident; a map of quartiles of U5MSR at country level, worldwide, is shown in figure 1. Excess female mortality was higher in Southeast Asia and to a lesser extent in the Eastern Mediterranean countries, while no clear pattern with country income was observed (data not shown).

GII varied from a minimum of 0.040 in Switzerland to a maximum of 0.767 in Yemen. The mean GII was 0.36 (SD 0.19) and the median was 0.37 (IQR 0.18–0.52). Mean GII decreased with increasing income groupings of countries, and the lowest levels of GII were registered in Europe.

**Figure 2:**
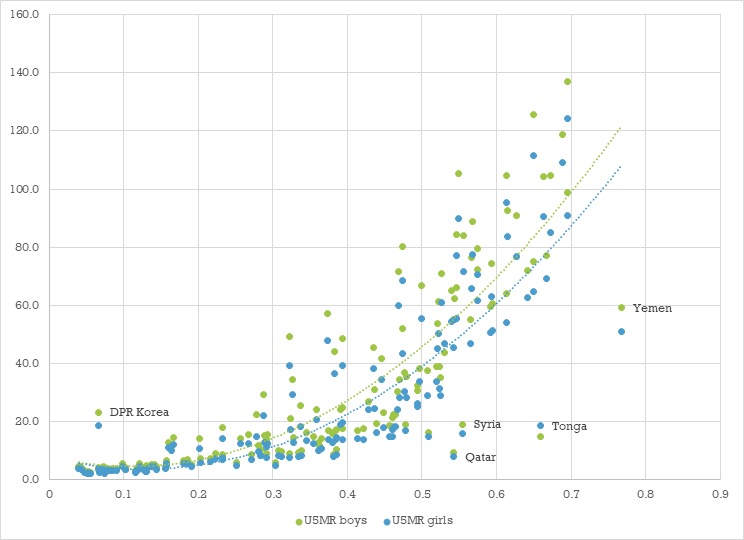
Scatterplot showing the association between the Gender Inequality Index (GII) on the X-axis and male (green) and female (blue) under-five mortality rate on the Y-axis, at country level, worldwide. Dotted lines show polynomial regression lines for boys and girls separately. U5MR, under-five mortality rate.

Results from the univariate linear regressions are presented in table 1. Gender inequality, acute respiratory infections and diarrhoea were significantly positively associated with both MCMR and FCMR in univariate analyses. On the other hand, the higher the GDP, the proportion of population accessing basic sanitation, and the proportion of births attended by skilled personnel, the lower were both the MCMR and the FCMR. The association of GII with MCMR and FCMR is plotted in figure 2.

**Table 1:** Crude and adjusted linear regression coefficient and relative 95% CI of factors potentially associated with male or female child mortality rates (MCMR or FCMR) and M:F under-five mortality ratio (U5MSR)

	N	MCMR*† coefficient (95% CI)	FCMR*† coefficient (95% CI)	U5MSR† coefficient (95% CI)	Excess female mortality coefficient (95% CI)
U5MSR					
Mean (SD)		–	–	1.20 (0.07)	
Median (IQR)		–	–	1.20 (1.17 to 1.23)	
Excess female mortality					
Mean (SD)		–	–		−0.60 (1.60)
Mean (IQR)		–	–		0 (−0.30 to 3.00)
GII					
Mean (SD)		–	–	0.36 (0.19)	
Median (IQR)		–	–	0.37 (0.18 to 0.51)	
Crude regression models					
GII 2015	156	5.28 (4.89 to 5.68)‡	5.37 (4.98 to 5.76)‡	−0.09 (-0.15 to –0.04)‡	0.20 (−1.38 to 1.42)
Acute respiratory infections*†	194	0.60 (0.57 to 0.63)‡	0.60 (0.58 to 0.63)‡	−0.004 (-0.001 to 0.001)	−0.08 (−0.21 to 0.05)
Diarrhoea*†	194	0.43 (0.40 to 0.46)‡	0.43 (0.40 to 0.47)‡	−0.004 (-0.008 to –3.5×10^–4^)§	−0.06 (−0.16 to 0.04)
GDP-per capita*	185	−0.69 (-0.74 to –0.63)‡	−0.69 (-0.75 to –0.64)‡	0.007 (1.2×10^–5^ to 0.01)§	0.10 (−0.07 to 0.27)
Proportion of population accessing at least basic sanitation*	192	−1.34 (−1.52 to –1.17)‡	−1.37 (-1.54 to –1.19)‡	0.03 (0.01 to 0.04)‡	0.32 (−0.06 to 0.70)
Proportion of births attended by skilled personnel*	180	−1.87 (−2.21 to –1.54)‡	−1.92 (−2.26 to –1.59)‡	0.06 (0.03 to 0.09)‡	0.11 (−0.57 to 0.80)
Multivariable regression mode					
GII 2015	140	1.49 (0.95 to 2.02)‡	1.75 (1.21 to 2.29)‡	−0.29 (−0.42 to –0.16)‡	3.25 (−0.28 to 6.78)
Acute respiratory infections*†	140	0.30 (0.22 to 0.37)‡	0.27 (0.20 to 0.35)‡	003 (0.01 to 0.05)§	−2.24 (−0.73 to 0.24)
Diarrhoea*†	140	0.07 (0.02/0.11)§	0.06 (0.02/0.11)§	5.9×10^–5^ (−0.01/0.01)	−0.01 (−0.42 to 0.46)
GDP per capita*	140	−0.08 (−0.14 to –0.01)§	−0.07 (−0.14 to –0.01)§	−0.005 (−0.02 to 0.01)	0.10 (−0.42 to 0.45)
Proportion of population accessing at least basic sanitation*	140	−0.16 (−0.29 to 0.04)§	−0.17 (−0.30. to –0.04)§	0.01 (−0.02 to 0.05)	0.90 (0.05 to 1.75)§
Proportion of births attended by skilled personnel*	140	−0.05 (−0.30 to 0.19)	−0.09 (−0.34 to 0.16)	0.05 (−0.01 to 0.11)	−0.79 (−2.41 to 0.83)

*Log-transformed values.

†Refers to under-five mortality rates per 1000 live births.

‡P values ≤0.001.

§P values≤0.005 and >0.001.

GDP, Gross Domestic Product; GII, Gender Inequality Index; FCMR, female child mortality rate; MCMR, male child mortality rate; U5SR, under-five mortality sex ratio.

The higher the GII of a country, the lower the U5MSR, implying that relatively less boys were dying as compared with girls (β-coefficient: −0.09 (95% CI −0.15 to –0.04), p<0.001): comparing perfectly gender unequal with perfectly gender equal countries, we would expect the U5MSR to decrease by 0.09 percentage points, which would correspond to nine boys’ but not girls’ lives spared per every 100 dead children. Diarrhoea, but not acute respiratory infections, was univariately negatively associated with U5SR. Conversely, GDP per capita, proportion of births attended by skilled personnel and proportion of population accessing sanitation were negatively associated with child mortality and positively associated with U5MSR, with an excess male child mortality increasing with the wealth of the country (β-coefficient 0.007 (95% CI 1.12×10^–5^ to 0.01, p=0.050)); increasing with the proportion of births attended by skilled personnel (β-coefficient 0.06 (95% CI 0.03 to 0.09, p<0.001)); and increasing with the proportion of population using at last basic sanitation services (β-coefficient 0.03 (95% CI 0.01 to 0.04, p<0.001)). None of the variables considered were univariately associated with excess female mortality as calculated by Alkema and colleagues.^15^ However, in the multivariable model, a borderline significant association between GII and excess female mortality was found (β-coefficient: 3.25 (95% CI −0.28 to –6.78), p=0.071).

Results from the multivariable analyses are presented in the bottom part of table 1. In adjusted models, all variables except the proportion of births attended by skilled personnel were associated with both male and female U5MSR in a statistically significant manner. However, only GII and U5MR from acute respiratory infections were significantly associated with U5MSR in the fully adjusted model: the average U5MSR was estimated to decrease between 0.42 and 0.16 percentage points comparing perfectly gender equal to perfectly gender unequal countries (β-coefficient −0.29 (95% CI −0.42 to –0.16), p<0.001). Conversely, the U5MR from acute respiratory infections was positively associated with U5MSR (table 1).

The adjusted association between GII and U5MSR was strengthened if MCMR was added to the model (β=−0.32 (95% CI −0.46 to –0.17, results not shown). After removal of outliers, that is, the only two countries with negative U5MSR (India and Tonga) and those with a U5MSR 2 SD above the mean (Mongolia and Viet Nam), all the multivariable associations with under-five mortality remained significant, including the association with U5MSR (β-coefficient=−0.23 (95% CI −0.35 to –0.11). After removal of eight outliers falling 2 SD below (Cote d’Ivoire, Guinea Bissau, Mauritania, Mongolia, Turkmenistan, Uganda and Zimbabwe) or above (Afghanistan, India, Niger, Pakistan) the mean, increasing GII of a country was significantly associated with an increasing excess female mortality within that country (β-coefficient=2.69 (95% CI 0.93 to 4.45, p=0.003).

**Figure 3:**
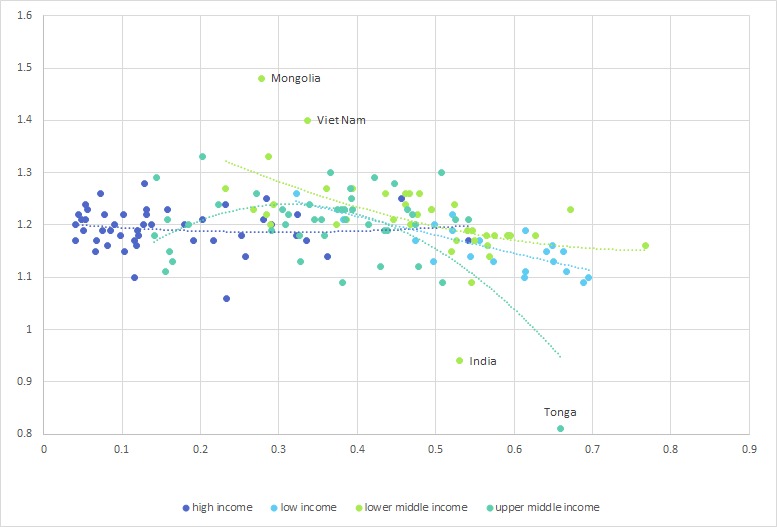
Scatterplot showing the association between the Gender Inequality Index on the X-axis and male to female under-five mortality rate on the Y-axis, at country level, worldwide, by country income categories. Dotted lines show polynomial regression lines for each category separately.

Effect modification by income groups and WHO regions was statistically significant or borderline when U5MSR was used as outcome measure (p=0.053 for GDP groups and p=0.050 for WHO regions). When excess female mortality was used as an outcome measure, only a modification by WHO regions was evident (p=0.258 for GDP groups and p<0.001 for WHO regions). Therefore, the analysis was repeated stratified by both variables with a significant interaction. Results for countries’ GDP groups are presented in table 2 and for WHO regions in online supplementary table 1. The association between GII and U5MSR was strong and statistically significant only in lower-income and upper-middle-income countries where the U5MSR was estimated to decrease by 0.42 and 46 percentage points comparing perfectly gender equal to perfectly gender unequal countries, respectively (β-coefficient −0.42 (95% CI −1.76 to –0.08), p=0.017 and β-coefficient=–0.46 (95% CI −0.76 to –0.15), p=0.004). Less strong, and borderline statistically significant was the same association in low-income countries (β-coefficient=−0.28 (95% CI −0.57 to 0.02), p=0.062), probably due to reduced power. Conversely, no association was apparent in high-income countries (β-coefficient=−0.09 (95% CI −0.26 to 0.09), p=0.321) (table 2). In figure 3, a scatterplot showing the association between GII and U5MSR by income categories is shown.

10.1136/bmjgh-2018-001028.supp1 Supplementary data

**Table 2:** Regression coefficients coming from multivariable models investigating the association of Gender Inequality Index (GII) with under-five mortality sex ratio (U5MSR) by income categories

	Low income N=21	Lower middle income N=40	Upper middle income N=43	High income N=36
U5MSR				
Mean (SD)	1.15 (0.04)	1.22 (0.08)	1.20 (0.08)	1.19 (0.04)
Median (IQR)	1.15 (1.13 to 1.19)	1.22 (1.18 to 1.26)	1.21 (1.19 to 1.24)	1.20 (1.17 to 1.22)
Excess female mortality				
Mean (SD)	−0.33 (2.25)	−0.08 (2.46)	−0.06 (0.92)	0.09 (0.26)
Median (IQR)	−0.30 (−1.80 to 0.90)	−0.10 (−0.90 to 0.20)	0.00 (−0.20 to 0.40)	0.00 (−0.10 to 0.10)
GII				
Mean (SD)	0.58 (0.08)	0.47 (0.12)	0.35 (0.12)	0.17 (0.11)
Median (IQR)	0.59 (0.52 to 0.65)	0.48 (0.38 to 0.55)	0.37 (0.27 to 0.44)	0.13 (0.09 to 0.25)
GII (U5SR)	−0.28 (−0.57 to 0.02)	−0.42 (−0•76 to –0.08)*	−0.46 (−0.76 to –0.15)*	−0.09 (−0.26 to 0.09)

The stratification by WHO zones was less easy to interpret, and in part hampered by reduction in power: the strongest and only significant negative association between GII and U5MSR was observed in the Western Pacific area (β-coefficient=1.06 (95% CI −1.78 to –0.33), p=0.010) (online supplementary table 1).

## Discussion

The results of this study suggest that, worldwide, gender inequality is associated with increased child mortality, and that girls seem to disproportionally suffer from this, particularly in lower-income and upper-middle-income countries. The higher the gender inequality in a country, as measured by GII, the higher the excess under-five female mortality measured either as under-five child mortality rate ratio, or as excess female mortality per 1000 live births.^15^ This finding reinforces the idea that gender inequality in a country perpetuates itself by directly impacting on child survival.

The present data confirm previous research findings^12 13^ showing that higher GII at country level is associated with increased child mortality; the higher the GII observed in a country, the higher the U5MR in both boys and girls. These associations are maintained even after adjusting for potentially biological and socioeconomic mediators/confounders. Moreover, increasing GII at country level is associated with a reduction in excess male over female child mortality. Again, this association is maintained after accounting for biological (U5MR from acute respiratory infections and diarrhoea), socioeconomic confounders (GDP per capita and access to sanitation) and maternal access to perinatal care (proportion of births attended by skilled personnel). Interestingly, U5MR from acute respiratory infections is associated with an increased excess in male over female mortality, probably reflecting boys’ biological disadvantage.^22^

The fact that the association between higher GII and male to female sex ratio of under-five mortality was further substantially strengthened once adjusted by male mortality, suggests that the association between measured gender inequality and excess female over male mortality is overall maintained across the spectrum of high and low U5MR. This is further confirmed by the borderline significant positive association between GII and the excess under-five female mortality as calculated by Alkema and colleagues^15^: comparing perfectly gender equal to perfectly gender unequal countries, we can expect the excess female mortality per 1000 live birth to increase by 3.25 percentage points.

Finally, the interaction between GII and country’s GDP plays an important role in the association between higher GII and child mortality as this association seems particularly driven in lower-income and upper-middle-income countries, and countries in the Western Pacific WHO region.

Previously, a number of pathways potentially explaining the ecological association between gender inequality and child mortality suggested^13^ female circumcision and infanticide, low birth weight of babies due to maternal undernutrition; domestic violence exposing babies to potentially fatal perinatal outcomes; low levels of women’s education preventing effective utilisation of healthcare; sexually and vertically transmitted infectious diseases; women’s lack of control over household economy; and child malnutrition.^13^ Only a few of these suggested pathways are likely to have an impact disproportionally on female compared with male children.

Under typical conditions, female infants and young children have an advantage in survival over boys of the same age.^22^ Thus, a male:female ratio >1 is not necessarily a sufficient standard for declaring that females do not experience disadvantage. Females could have mortality rates that are lower than those of males but still not as low as would be expected given girls’ genetic and biological survival advantage.^23^ In certain historical populations where discrimination against female children was believed to be negligible, such as in European populations with comparable mortality rates, sex differentials in infant, child and under-five mortality increased as the level of mortality declined.^24^ However, the fact that our results were reproduced when using the excess female mortality as computed by Alkema and colleagues^15^ which accounts for this reinforces our inference on a true association between GII and excess under-five female mortality.

This study supports a global pattern which was already observed in local contexts.^25^ Our findings that gender inequality may be related to a social penalty,^10 26^ which in many cases outweighs the biological survival advantage of female infants and children,^27^ can potentially be explained by two pathways: (1) the direct relationship between gender inequality and mortality for female infants and children and (2) the indirect relationship between gender inequality and outcomes for mothers. First, because of gender inequality resulting from sexist ideology which values boys and devalues girls, young girls are at greater risk of mortality through diminished access to health-promoting resources as well as through heightened exposure to health risks.^27 28^ For example, in India girls have lower odds than boys of receiving facility-based curative and preventive care^29^ and vaccinations^30^ and are simultaneously vulnerable to female infanticide and circumcision,^25^ resulting in socially rather than biologically-rooted, sex differences in infant and child mortality rates.^31^ Second, gender inequality may contribute to sex differences in infant and child mortality through its impact on mothers. Maternal undernutrition, exposure to violence and lack of access to education results in children who are more vulnerable to negative health outcomes through both biological and social mechanisms such as susceptibility to communicable and non-communicable diseases as well as decreased access to preventive health practice.^32^ The increased impact on girls’ survival chances is again explained by the fact that mothers of daughters are valued less than mothers of sons and hence are more often exposed to the aforementioned risk factors due to the responsible cultural preferences.^26 33^ This may result in negating the biological survival advantage of female children over their male counterparts. The finding in this study that only maternal access to perinatal care (approximated by the proportion of births attended by skilled personnel) is not associated with U5MSR in the adjusted model, and that conversely higher under-five mortality from acute respiratory infection is associated with an excess male over female mortality, suggests that relevant maternal factors should be explored beyond the perinatal care field.

The stronger association between the GII and U5MSR in low-income and middle-income countries suggests that the intersection between a country’s level of economic growth and gender inequality may generate a disproportionate disadvantage only for female infants and young children. This finding is particularly alarming^9^ if we consider that the gender gap systematically decreases with levels of economic development.^28 34 35^ Moreover, it may also imply that processes of capitalist and neoliberal transition that are often in progress in middle-income countries bear a disproportionate mortality disadvantage for young girls. In more generalised terms, this suggests that structural mechanisms of socioeconomic stratification and gender inequality operate simultaneously as drivers of survival decrease for both boys and girls. However, their overall impact on child mortality is not simply the sum of their separate effects but rather the unique outcome of the way they intersect with each other and with individual social positions since the outcome is different for girls than it is for boys.^36^ Hence, an intersectional approach is necessary for the development of a better understanding of gendered inequalities in child mortality and for any relevant policy formation and evaluation. Interventions that focus on increasing a country’s wealth in order to tackle child mortality should adopt a gender-sensitive perspective in order to benefit those who bear the double burden of gender inequality and poverty.

A key limitation of this study is ecological fallacy, where conclusions cannot be drawn about individuals from the aggregated data. This means that we cannot conclude that, within each country, the excess female mortality is observed where the gender inequity is higher. However, gender inequality is an aggregate measure calculated at country level and should be assumed to apply to the entirety of the country population. In addition, the GII is a relatively new indicator and is not necessarily a representative measure of gender inequality. Moreover, although the rationale that gender inequality in mortality is a manifestation of the overall power imbalance experienced by people within a patriarchal system allows us to put our results in the context of societal power relations, the current study does not investigate the particular mediation mechanisms responsible for the observed inequality. Future research should explore this and ideally within specific national contexts. There is also the potential for systematic differences between countries in recording child mortality and the frequency of it. Many studies suggest a disproportionate amount of missing data of female deaths in certain low-income countries as many families do not report them.^37^ It is impossible to estimate the effect of unmeasured or residual confounding effect on this association, nor data quality. Finally, using linear regression models, some non-linear effects of the associations studied might have been missed.

## Conclusions

This study adds to the growing body of literature suggesting that gender inequality results in negative societal health. The results from this paper suggest that gender inequality in society is able to perpetuate itself as the more gender unequal a society is, the more girls are penalised, in terms of their survival chances. In order to decrease child mortality in general, global policy should focus on reducing gender inequality surrounding reproductive health, women’s political empowerment, educational attainment and participation in the workforce. Only until these measures are addressed will the detrimental transgenerational effects of gender inequality on child mortality, and particularly, girls’ child mortality, be alleviated.
