# Supplementary material for: Girls’ hidden penalty: analysis of gender inequality in child mortality with data from 195 countries
Source: BMJ Glob Health. 2018 Oct 30;3(5):e001028. doi: 10.1136/bmjgh-2018-001028 (PMC6231099; doi:10.1136/bmjgh-2018-001028)
Supplement: Supplementary data [file bmjgh-2018-001028supp001.pdf]

Supplementary Table 1: Regression coefficients coming from multivariable models investigating the association with under-5 mortality sex ratio (U5MSR) by WHO regions

	Africa N=34	Americas N=28	South-East Asia N=9	Europe N=40	Eastern Mediterranean N= 14	Western Pacific N=15
U5MSR						
mean (SD)	1•17 (0•04)	1•23 (0•04)	1•18 (0•10)	1•22 (0•05)	1•15 (0•05)	1•20 (0•15)
median (IQR)	1•8 (1•14-1•20)	1•23 (1•21-1•25)	1•21 (1•15-1•22)	0•21 (0•18-1•24)	1•17 (1•11-1•20)	1•19 (1•17-1•26)
Excess female mortality						
Mean (SD)	-0.75 (1.79)	0.03 (0.56)	1.51 (4.15)	-0.20 (0.83)	0.98 (1.49)	-0.24 (0.90)
Median (IQR)	-0.40 (-1.70/0.40)	0.10 (-0.20/0.30)	0.10 (-0.60/2.10)	0.00 (-0.10/0.00)	0.50 (0.20/1.10)	0.00 (-0.30/0.10)
GII						
mean (SD)	0•55 (0•09)	0•38 (0•10)	0•44 (0•08)	0•18 (0•10)	0•46 (0•16)	0•33 (0•18)
median (IQR)	0•55 (0•52-0•62)	0•38 (0•32-0•46)	0•47 (0•37-0•50)	0•16 (0•110•27)	0•50 (0•29-0•55)	0•34 (0•16-0•47)
GII 2015 (U5MSR)	-0•16 (-0•39/0•07)	0•19 (-0•16/0•55)	0•98 (-0•53/2•49)	-0•18 (-0•48/0•12)	-0•05 (-0•48/0•38)	-1•06 (-1•78/-0•33)*
GII 2015 (excess fem. mort)	2.27 (-0.10/12.62)	-5.29 (-10.7/0.12)	-30.8 (-85.5/24.7)	2.34 (-1.16/5.84)	-0.27 (-13.5/13.0)	2.16 (-5.59/9.91)

U5MSR: under 5 mortality sex ratio; GII: Gender Inequality Index; SD: standard deviation; IQR: inter quartile range
